# Impaired Memory for Instructions in Children with Attention-Deficit Hyperactivity Disorder Is Improved by Action at Presentation and Recall

**DOI:** 10.3389/fpsyg.2017.00039

**Published:** 2017-01-24

**Authors:** Tian-xiao Yang, Richard J. Allen, Joni Holmes, Raymond C. K. Chan

**Affiliations:** ^1^Neuropsychology and Applied Cognitive Neuroscience Laboratory, CAS Key Laboratory of Mental Health, Institute of PsychologyBeijing, China; ^2^School of Psychology, University of LeedsLeeds, UK; ^3^MRC Cognition and Brain Sciences UnitCambridge, UK; ^4^University of Chinese Academy of Sciences (CAS)Beijing, China

**Keywords:** ADHD, working memory, following instructions, enactment, action benefits

## Abstract

Children with attention deficit hyperactivity disorder (ADHD) often fail to comply with teacher instructions in the classroom. Using action during presentation or recall can enhance typically developing children’s abilities to complete multi-step instruction sequences. In this study, we tested the ability to following instructions in children with ADHD under different conditions to explore whether they show the same beneficial effects of action. A total of 24 children with ADHD and 27 typically developing children either listened to or viewed demonstrations of instructions during encoding, and then either verbally repeated or physically performed the sequences during recall. This resulted in four conditions: spoken-verbal, spoken-enacted, demonstration-verbal, and demonstration-enacted. Children with ADHD were significantly impaired in all conditions of the following instructions task relative to the typically developing group. Both groups showed an enacted-recall advantage, with superior recall by physical performance than oral repetition. Both groups also benefitted from demonstration over spoken presentation, but only when the instructions were recalled verbally. These findings suggest that children with ADHD struggle to complete multi-step instructions, but that they benefit from action-based presentation and recall in the same way as typically developing children. These findings have important implications for educators, suggesting that motor-based methods of instruction-delivery might enhance classroom learning both for children with and without developmental disorders.

## Introduction

Attention deficit hyperactivity disorder (ADHD) is a common developmental disorder associated with elevated symptoms of hyperactivity/impulsivity and inattention. Symptoms typically emerge in childhood and continue into adolescence and adulthood (Simon et al., 2009). The economic impact of the disorder is substantial due to life-long psychosocial and psychiatric burden (Castellanos and Proal, 2012) that arises from persistent problems in educational attainment and behavioral control (Loe and Feldman, 2007). Observational studies and those relying on behavior ratings suggest that children with ADHD comply less with classroom instructions and are more prone to breaking rules than their typically developing peers (Abikoff et al., 2002). Being able to encode, maintain and implement instructions underpins the ability to complete individual learning activities in the classroom that are important for knowledge acquisition (Gathercole and Alloway, 2008). Difficulties completing classroom instructions may therefore underpin some of the longer-term educational problems linked with ADHD (Loe and Feldman, 2007). The aim of this study was to provide the first direct assessment of following instruction (FI) skills in children with ADHD, and to explore whether their abilities to successfully implement instructions could be enhanced by action during the presentation and recall of instruction sequences in the same way that it can for typically developing children and adults (Gathercole et al., 2008; Yang et al., 2014, 2015, 2016; Allen and Waterman, 2015; Jaroslawska et al., 2016a).

The ability to complete complex multi-step sequences of instructions is important for many everyday tasks ranging from following parent or teacher instructions to guide learning and development in childhood through to understanding how to use new systems in the workplace in adulthood. Little is currently known about the cognitive and neural systems underpinning this fundamental skill, but it likely draws on a variety of higher-order cognitive control functions that allow us to remember what has to be done (e.g., monitoring), attend to the task at hand (e.g., sustained attention), make plans to sequential actions (e.g., planning), inhibit the tendency to execute the current action immediately (e.g., inhibition), and switch flexibly between different steps to reach an end goal (e.g., switching). Although there have been no direct investigations into the relationship between other executive function skills and FI abilities, greater problems in daily activities relying on FI are reported in populations with poor executive control (e.g., problems following cooking recipes and medication schedules are consistently reported for aging populations) (Cahn-Weiner et al., 2000; Bell-McGinty et al., 2002). Neuroimaging studies also suggest that planning and executing actions involves multiple neural networks associated with executive functioning including supplementary and primary motor areas, cerebellum, basal ganglia, and prefrontal cortex (Hommel et al., 2016).

In addition, recent research suggests that working memory, the cognitive system responsible for the temporary maintenance and processing of information (e.g., Baddeley and Hitch, 1974; Cowan, 2005; Baddeley, 2012), might support FI. In adults, the ability to perform instructions is impaired by concurrent tasks designed to disrupt different aspects of working memory (Yang et al., 2014, 2016). In children, the ability to follow spoken instructions is related to performance on working memory tasks (Engle et al., 1991; Gathercole et al., 2008; Jaroslawska et al., 2016b), with pronounced difficulties in FI observed in those with poor working memory (Gathercole et al., 2006; Alloway et al., 2009). These impairments likely arise as a consequence of the loss of task relevant information from working memory (e.g., Gathercole and Alloway, 2008) and have been related to other problems including low academic achievement, attentional difficulties and wider problems in executive function (Alloway et al., 2009; Holmes et al., 2014).

Recent research has begun to explore ways in which FI might be enhanced. A typical FI paradigm involves remembering series of multi-step actions (e.g., *Touch the white bag and then pick up the yellow ruler*), often involving action-based processing at either encoding or retrieval stage enhanced the performance. Action-related benefits at encoding emerge when participants are asked to perform instructions themselves during spoken presentation (Charlesworth et al., 2014; Allen and Waterman, 2015). Observing action during encoding can also facilitate recall. Improved recall is observed following the demonstration of instruction sequences as performed in person by the experimenter (Wojcik et al., 2011) or shown as on-screen demonstrations (Yang et al., 2015). Recall accuracy is consistently enhanced when instructions are recalled through physical action relative to spoken repetition (Koriat et al., 1990; Yang et al., 2014, 2016; Allen and Waterman, 2015). The benefits of action-based manipulations at encoding and recall appear to be at least somewhat interactive, with larger facilitatory effects of enactment during presentation emerging when participants verbally recall instructions, compared to when they enact at recall (Allen and Waterman, 2015; Yang et al., 2015). This suggests some commonalities in the source of these varying encoding- and recall-based action effects. While the precise mechanisms are yet to be established, this may involve increased engagement with additional forms of coding, including visuospatial and/or motoric forms of representation, which provide a richer and more robust representation relative to those normally created to serve the verbal recall of spoken sequences.

The aims of the current study were: (i) to provide the first direct assessment of FI skills in children with ADHD relative to a typically developing group and (ii) to investigate whether action during the presentation of the instructions in the form of on-screen demonstration, or physical action during recall, could enhance performance in both groups. We used a paradigm based on that reported by Gathercole et al. (2008) that requires children to remember increasingly longer sequences of instructions. To investigate the impacts of action-based encoding and response manipulations in the current study, children either listened to or viewed the sequences of instructions during encoding, and then either verbally repeated or physically performed them during recall (i.e., four conditions: spoken-verbal, spoken-enacted, demonstration-verbal, and demonstration-enacted).

We predicted that FI would be impaired in the ADHD group relative to the control group across all four conditions of the FI task for several reasons. First, deficits in working memory are a core feature of the disorder (Martinussen et al., 2005) and typically extend to a wide range of other executive functions including attentional switching (e.g., Oades and Christiansen, 2008), sustained attention (Rubia et al., 2009), planning (e.g., Solanto et al., 2007), and response inhibition (e.g., Bledsoe et al., 2010). Second, many distinct large-scale neural networks, including the executive control circuit based within the fronto-parietal network, dorsal and ventral attentional networks, distinct visual and motor networks, and the default network, may be affected in ADHD (Sonuga-Barke and Castellanos, 2007; Castellanos and Proal, 2012; Cortese et al., 2012). Given the complex multi-modal nature of FI, it is plausible that disruptions in any one or combination of these cognitive or neural networks may lead to difficulties.

Due to both the heterogeneity in cognitive problems associated with ADHD and the complex nature of FI no specific predictions were made regarding differences in the magnitude of impairments across FI conditions for the ADHD group. Different conditions might be expected to draw on different cognitive processes. For example, the spoken and verbal conditions might rely more heavily on language skills than the action-based conditions, which may in turn draw more on visuospatial resources, motor control, and the ability to construct motor representations. The language problems commonly reported in ADHD (Martinussen and Tannock, 2006) might be expected to affect conditions involving verbal processing (e.g., those involving spoken presentation and verbal recall), while difficulties in motor processing (Halperin et al., 2008; Rapport et al., 2009) and visuospatial working memory (Martinussen et al., 2005) that are also commonly found in ADHD might influence performance in the action-based conditions. Problems with attention are also likely to affect performance across the conditions in various ways. For example, difficulties switching attention (Oades and Christiansen, 2008) might affect FI conditions involving different encoding and retrieval modalities (i.e., spoken-enacted recall and demonstration-verbal recall), while problems sustaining attention (Rubia et al., 2009) might have a broader negative influence across all conditions.

Second, we predicted improved memory accuracy following demonstrated encoding and using enacted recall for both ADHD and typically developing children. For typically developing children, this prediction was based on previous observations (Gathercole et al., 2008; Jaroslawska et al., 2016a). How might children in the ADHD group respond to these different encoding and response manipulations? The action-based effects in FI tasks may be relatively automatic and non-strategic in nature (e.g., Wojcik et al., 2011; Charlesworth et al., 2014; Yang et al., 2014, 2016). The possibly automatic and non-strategic boosts that such manipulations might bring, as well as their potential to increase participant engagement in the task (thus reducing the probability of attentional lapses, which children with ADHD normally suffer from), mean that the ADHD group might also demonstrate beneficial effects of action. Given the range of spatial, motoric, and linguistic processes likely engaged by the different experimental conditions, and evidence that children with ADHD might be impaired in each of these abilities (Martinussen et al., 2005; Martinussen and Tannock, 2006; Halperin et al., 2008; Rapport et al., 2009), we did not formulate a clear prediction regarding whether the ADHD group would show equivalent or reduced action-based effects relative to those observed in typically developing children. This therefore remained an open question, with findings likely to inform theory and practice regarding FI and ADHD.

## Materials and Methods

### Participants

Children diagnosed with ADHD were recruited from a local hospital in Beijing. The inclusion criteria were: (1) diagnosed with ADHD according to DSM-IV criteria (American Psychiatric Association, 1994) by pediatric psychiatrists and (2) IQ > 80 on a short version of the Wechsler Intelligence Scale for Children (in Chinese) (Gong and Cai, 1993). Exclusion criteria were: (1) comorbid anxiety or conduct disorder and (2) known neurological disorder (e.g., tic disorder).

A total of 24 ADHD children (16 boys and eight girls, mean age = 8.32, *SD* = 1.44, age ranged from 6.60 to 11.00 years) were recruited. Of the 24 children with ADHD, 10 were diagnosed with the inattentive subtype, 12 with the combined subtype, and two with the hyperactive/impulsive subtype. At the time of testing, 18 out of 24 children were taking medication for their ADHD symptoms. Eleven children were taking Methylphenidate Hydrochloride Prolonged-Release tablets (mean dosage = 21.27 mg/day, *SD* = 7.28). Seven children were taking atomoxetine hydrochloride Capsules (mean dosage = 34.29 mg/day, *SD* = 9.76). Those who were medicated continued to take their medication throughout the study.

Twenty-seven typically developing children (16 boys and 11 girls, mean age = 8.51, *SD* = 1.29, age ranged from 6.40 to 11.30 years) were recruited from local communities in Beijing and Weifang, China. None had an ADHD diagnosis. The mean IQ was 106.04 (9.59) for the ADHD group and 109.78 (10.62) for the typically developing group. Standard and Bayesian *t*-tests (JASP Team, 2016) revealed no significant group differences in age (*t*_(49)_ = -0.47, *p* = 0.640, *Bayes Factor (BF)* = 0.31), gender (χ_(1)_^2^ = 0.30, *p* = 0.585, *BF* = 0.32) or IQ (*t*_(48)_ = -1.30, *p* = 0.201, *BF* = 0.57).

### Procedure

Experimental procedures were approved by the Institutional Review Board of the Institute of Psychology, Chinese Academy of Sciences and the Peking University Sixth Hospital. The experiment was carried out in accordance with the approved guidelines. Informed consent was obtained from parents/carers at the start of the testing.

Children in the ADHD group were tested individually in a quiet room in a psychiatric hospital. Those in the typically developing group were tested individually in a quiet room in a university. The tests were administered in a single 90-min session in the following fixed order: IQ, Opposite World, a prospective memory task (a task carried out for a different study, involving a game requiring remembering to “feed a cat” at a specific time or event occurrence), Walk Don’t Walk, and the FI span task. The FI tasks were always administered last, but the sequence of conditions was counterbalanced across children using a Latin square design.

### Design

A 2 × 2 × 2 mixed design was used. The within-subjects factors were presentation (spoken vs. demonstrated instructions) and recall type (verbal vs. enacted recall). The four conditions are spoken-verbal, spoken-enacted, demonstration-verbal, and demonstration-enacted. The between-subjects factor was group (ADHD vs. typically developing). The order of the four within-subject conditions was counterbalanced between the participants, and the same counterbalanced order was implemented for the two groups.

### Materials

#### Sustained Attention and Response Inhibition

Two tasks were administered from the Chinese version of the Test of Everyday Attention for Children (Manly et al., 2001; Chan et al., 2008).

##### Walk Don’t Walk

This task tapped a child’s ability to sustain attention and inhibit a prepotent response. The child is given a sheet showing paths made up of footprints and has to dot the next footprint on the path with a marker pen when they hear a frequently occurring “go” sound. The child is instructed not to dot the next footprint when an occasional “no go” sound is played. There were 20 trials, resulting in a score ranging from 0 to 20.

##### Opposite World

This task measures attentional control and response inhibition. The child is shown an array of 24 randomly arranged digits (either digit 1 or 2) on a sheet. In a “same world” trial, the child names the digits as quickly as possible. In an “opposite world” trial, he/she names the alternate digit to the one that is visually presented (i.e., say “one” to digit “two”, and “two” to digit “one”). If a child makes an error, he/she is required to correct it. The extra time cost of the correction is taken into account. The average time (in ms) to complete the same and opposite world trials was scored.

#### Intelligence Quotient (IQ)

IQ scores for the ADHD group were provided by the hospital using the Chinese version of the full Wechsler Intelligence Scale for Children (Gong, 1992), which was administered within 6 months of this study. IQ scores were obtained for the typically developing group using the Chinese short version of the Wechsler Intelligence Scale for Children (Gong and Cai, 1993), which correlates highly with the full scale IQ test (*r* = 0.95).

#### The ADHD Rating Scale-IV

The home version of the Chinese ADHD Rating Scale–IV (ADHD RS-IV) was used to evaluate children’s inattention, hyperactivity and impulsive behaviors (Su et al., 2012). The scale comprised a total of 18 items, and each item was rated on a four-point scale (1 = none, 2 = occasionally, 3 = often, 4 = always) by the parent of the child. It has two subscales (“inattention” and “hyperactivity-impulsivity”), and each contained nine items and had a score range from 9 to 36.

#### Following Instruction Span Task

This task was adapted from a previous study with young adult participants (Yang et al., 2015). A typical instruction comprised a series of pairs of different movements and colorful stationery objects dispersed on a table. There were five types of movements (touch, push, drag, spin, pick up, put it into), six small objects (a yellow ruler, a blue ruler, a white eraser, a green eraser, a red pencil, and a black pencil), and six containers (a yellow basket, a white basket, a blue folder, a green folder, a red bag, and a black bag). For example, a three-action instruction sequence might involve “pick up the white eraser then put it into the blue folder, and touch the green folder.” Each condition involved six blocks of trials, with each block containing six instructional trials of the same number of actions. The instructions in the first block contained only one action (e.g., *spin the green eraser*), the second block contained two actions (e.g., *push the red pencil and touch the yellow ruler*), and so forth. Four sets of instructional lists were constructed (one for each condition).

Spoken instructions were recorded by a Native Chinese female speaker at a moderate speed of approximately 350 ms per word. Demonstration of instructions involved use of video clips showing a series of hand movements upon objects. In both the spoken and demonstration conditions, the durations for the presentation of sequences of 1–6 actions were 3, 5, 8, 11, 13, and 16 s, respectively.

**FIGURE 1:**
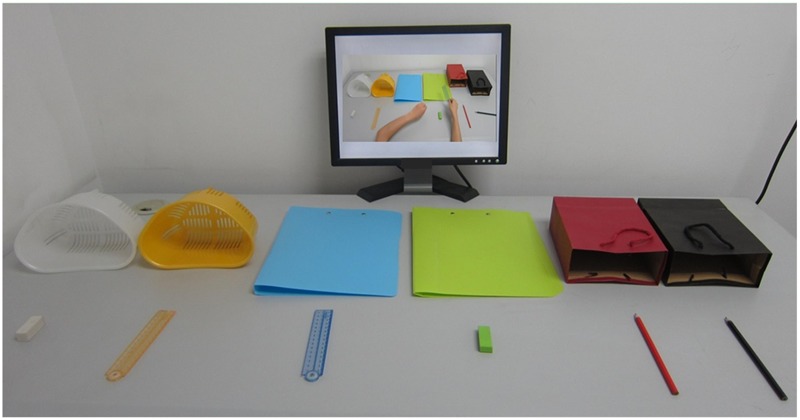
**The setting of the visual display for the demonstration condition.** Figure reproduced from Yang et al. (2015).

Each child completed all four conditions. In each condition, a child sat at a desk facing the stationary objects and a computer monitor for displaying instructions (see **Figure 1**). The experimenter sat at another desk away from the child and controlled the delivery of instructions. The experimenter first introduced the task, followed by a practice of object naming and operation to ensure that the child understood the task requirements. Children were told not to repeat the instructions aloud, touch, operate or move the objects during encoding. In a typical trial of the spoken instruction, the experimenter first signaled the child to get ready, and then played the instructions through speakers. In a trial of the demonstration condition, children viewed silent video clips of actions. In all conditions, a blank screen would appear at the end of the trial, indicating the recall phase. Children then either repeated the instructions (verbal recall) or performed the actions (enactment recall). Children started from one-action instruction and progressed to the next span if they correctly recalled four out of six trials at a given sequence length. Performance on these tasks was measured in terms of action scores, with a score recorded as correct when the movement, color and object of an action “chunk” were recalled in the correct serial position (range 0–126).

## Results

### Attention

Descriptive statistics and group comparisons are reported in **Table 1**. The ADHD group were significantly poorer on both measures of attention compared to the typically developing group. There were no significant differences in performance between the medicated and unmedicated children [Walk Don’t Walk, Means (*SD*) was 8.83 (5.15) for the unmedicated ADHD group, and 9.56 (4.51) for the medicated group, *t*_(22)_ = -0.33, *p* = 0.746, Cohen’s *d* = 0.16; Opposite Worlds, Means (*SD*) was 34.63 (15.01) for the unmedicated ADHD group, and 35.18 (6.80) for the medicated ADHD group, *t*_(22)_ = -0.13, *p* = 0.902, Cohen’s *d* = 0.06]. Bayesian *t*-tests favored the absence of group differences on these measures (Walk Don’t Walk *BF* = 0.43; Opposite Worlds *BF* = 0.41).

**Table 1:** Measures of sustained attention, and response inhibition in children with ADHD and typically developing children.

	ADHD (*N* = 24)		Typical (*N* = 27)		Group comparison			Bayesian *t*-test
	Means	*SD*	Means	*SD*	*t*	*p*	Cohen’s *d*	*BF*
Walk Don’t Walk	9.38	4.58	14.30	2.74	4.59	<0.001	1.31	891
Opposite world	35.05	9.12	25.89	9.22	3.56	0.001	1.02	36

The behavior rating scale data is presented in **Table 2**. The ADHD group were rated as significantly more inattentive and hyperactive/impulsive than the typically developing group, with higher overall scores that were indicative of a greater number of ADHD symptoms.

**Table 2:** Scores of rating scales in children with ADHD and typically developing children.

	ADHD (*N* = 17)^a^		TD (*N* = 21)^a^		Group comparison			Bayesian *t*-test
	Means	*SD*	Means	*SD*	*t*	*P*	Cohen’s *d*	*BF*
ADHD RS-IV total	1.67	0.25	0.90	0.40	7.26	<0.001	2.43	>1000
ADHD RS-IV IA	1.97	0.33	1.12	0.41	6.87	<0.001	2.32	>1000
ADHD RS-IV HI	1.38	0.47	0.79	0.45	3.88	<0.001	1.32	62

*ADHD, attention deficit hyperactivity disorder; TD, typically developing children; ADHD RS-IV, ADHD Rating Scale-IV; IA, Inattention subscale; HI, hyperactivity impulsivity. ^a^Questionnaires were not returned for seven of the ADHD group and six of the typically developing group.*

### Following Instructions

**FIGURE 2:**
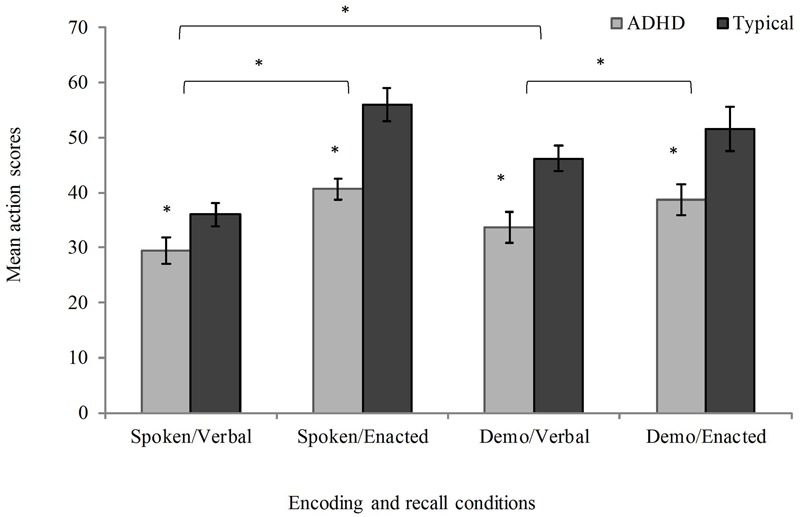
**Mean action scores (max 126; error bars represent standard errors) as a function of presentation and recall type in children with attention deficit hyperactivity disorder (ADHD) and typical developing children; Demo = Demonstration.** ^∗^*p* < 0.05.

There were no significant differences in performance on any of the FI tasks between the ADHD subtypes (inattentive vs. combined), or between those with ADHD who were medicated and those who were unmedicated (see Supplementary Material). The data were therefore collapsed into a single ADHD group in all subsequent analyses. Descriptive statistics and group comparisons in action scores for all conditions of the FI span task are presented in **Figure 2**.

To investigate whether there were group differences in the impact of action at presentation or recall, a 2 × 2 × 2 mixed ANOVA was run with presentation condition (spoken, demonstration) and recall modality (verbal, enactment) as within-subjects factors and group (ADHD, typically developing children) as a between-subjects factor. This was followed by *t*-tests comparing the typically developing and ADHD group in each FI condition. The ANOVA test indicated a significant main effect of group, with the ADHD group showing reduced recall performance compared to the typically developing group (*F*_(1,49)_ = 16.72, *p <* 0.001, $\eta_{p}^{2}$ = 0.25). The *t*-tests revealed significant deficits in each FI condition (spoken presentation/verbal recall, *t*_(49)_ = 2.04, *p* = 0.047, Cohen’s *d* = 0.58; spoken presentation/enacted recall, *t*_(49)_ = 4.26, *p* < 0.001, Cohen’s *d* = 1.22; demonstration presentation/verbal recall, *t*_(49)_ = 3.49, *p* = 0.001, Cohen’s *d* = 0.99; demonstration presentation/enacted recall, *t*_(49)_ = 2.58, *p* = 0.013, Cohen’s *d* = 0.74).

In terms of action-based advantages, the mixed ANOVA showed a significant main effect of recall type, with superior recall by enactment than oral repetition (i.e., enacted-recall advantage, *F*_(1,49)_ = 44.98, *p <* 0.001, $\eta_{p}^{2}$ = 0.48). The main effect of presentation was not significant (*F*_(1,49)_ = 1.58, *p =* 0.215, $\eta_{p}^{2}$ = 0.03), but there was a significant interaction between presentation and recall (*F*_(1,49)_ = 13.77, *p =* 0.001, $\eta_{p}^{2}$ = 0.04). Further *t*-tests revealed an advantage for demonstration over spoken presentation when the instructions were recalled verbally (*t*_(50)_ = 3.81, *p* < 0.001, Cohen’s *d* = 0.57), but not when they were enacted at recall (*t*_(50)_ = -1.36, *p =* 0.179, Cohen’s *d* = 0.19). The benefits of enactment at recall were present for both spoken (*t*_(50)_ = 8.18, *p <* 0.001, Cohen’s *d* = 1.19), and demonstrated instruction sequences (*t*_(50)_ = 2.30, *p =* 0.025, Cohen’s *d* = 0.32), with substantially larger effect sizes for spoken instructions. There was no significant interaction between group and presentation type (*F*_(1,49)_ = 0.29, *p =* 0.596, $\eta_{p}^{2}$ = 0.01), or group and recall modality (*F*_(1,49)_ = 2.14, *p =* 0.150, $\eta_{p}^{2}$ = 0.04), nor a significant three-way interaction between group, presentation, and recall type (*F*_(1,49)_ = 2.23, *p =* 0.142, $\eta_{p}^{2}$ = 0.04).

In addition to the traditional frequentist analysis above, a 2 × 2 × 2 mixed Bayesian ANOVA was also carried out (JASP Team, 2016), in order to assess the strength of evidence for each main effect and interaction. The best model (*BF* > 1000 versus the intercept-only model, ±3.03%) included two main effects (group and recall) and the interaction between presentation and recall. Inclusion of effects of recall (*BF* > 1000) and group (*BF* = 136) were strongly favored, as was the interaction between presentation and recall (*BF* = 41). The analysis did not support the inclusion of the presentation factor (*BF* = 0.31), or the interactions between presentation and group (*BF* = 0.26), recall and group (*BF* = 0.52), or the three-way interaction (*BF* = 0.56). Bayesian *t*-tests indicated evidence for differences between typically developing and ADHD groups in each condition (spoken presentation/verbal recall, *BF* = 1.49; spoken presentation/enacted recall, *BF =* 235.40; demonstration presentation/verbal recall, *BF* = 27.32; demonstration presentation/enacted recall, *BF* = 3.93). These outcomes are therefore in line with those obtained using the null hypothesis testing analysis of variance.

## Discussion

This study investigated FI in children with ADHD and explored whether action-based presentation and recall improved performance equally in typically developing children and those with ADHD. The ADHD group were impaired in FI compared to the typically developing group irrespective of the mode of presentation or recall. They did, however, display similar patterns of action-related benefits as the typically developing group. Both groups showed an enacted-recall advantage, with superior recall of instructions by enactment than oral repetition. There were also benefits of demonstration at presentation over spoken presentation, but only when recall was verbal.

Overall, and in line with our prediction, children with ADHD were impaired in all conditions of the FI paradigm. As the FI task involves multiple modalities and recruits working memory resources (Yang et al., 2014, 2016), this general pattern of impairment across conditions may at least partly reflect an impairment in the executive control aspect of working memory that is common in ADHD (Holmes et al., 2014). It may also reflect broader problems in higher-order cognitive control functions (Solanto et al., 2007; Oades and Christiansen, 2008; Rubia et al., 2009; Bledsoe et al., 2010) and related dysfunctions of executive-control and attention-related brain networks (Castellanos and Proal, 2012; Cortese et al., 2012). Consistent with this idea, the ADHD group had problems on the executive function tasks measuring sustained attention and inhibition. A combination of poor executive control and impaired working memory in children with ADHD may constrain their ability to encode, hold in mind and subsequently act upon sequences of commands.

An advantage was found for both groups for instructions that were enacted at recall. This is consistent with previous literature showing robust benefits of action at recall following the spoken presentation of instructions (Gathercole et al., 2008; Allen and Waterman, 2015; Yang et al., 2015, 2016). In terms of the presentation of instructions, there were no overall differences in performance when instructions were delivered as oral commands or as demonstrated actions. There was, however, an interaction with recall type, with an advantage for demonstration at presentation when instructions were recalled by spoken repetition, but not when they were enacted. This interactive relationship between demonstrated encoding and enacted recall benefits is broadly in line with recent outcomes from research with young adults (Yang et al., 2015), and also fits with studies examining the effects of self-enactment during encoding (Allen and Waterman, 2015), suggesting some commonalities in the processes operating during encoding in each case. In the present study, demonstration may facilitate verbal recall through the provision of additional visual, spatial, and motor information. In contrast, planning for enacted recall may generate effective action-based representations, meaning there was reduced additional gain to be made from the provision of this additional spatial-motor information at presentation (see Allen and Waterman, 2015, for a related explanation of self-enactment effects during encoding). According to the common coding theory of action planning and action perception (Prinz, 1997), the action-based presentation provided by demonstration may resemble the representation formed by active action planning for the enacted recall of spoken instructions. This would explain the similar levels of performance in enactment recall performance of spoken and demonstrated instructions observed in the present study.

Importantly, both the typically developing and ADHD groups showed equivalent performance benefits as a result of demonstration and enacted recall. Particular deficits in ADHD for both visuospatial working memory (e.g., Martinussen et al., 2005) and motor processing (e.g., Barkley, 1997; Pitcher et al., 2003) have previously been observed, which might be expected to mitigate against this group benefitting in conditions that may reflect these forms of coding. Although the deficits in the ADHD group appear to be numerically slightly smaller for the condition involving the least action-based processing (spoken presentation/verbal recall), the ADHD group displayed a similar overall profile of performance to the typical children. Recent evidence suggests the enacted recall advantage may be independent of working memory components as set out in the Baddeley (1986) tripartite model (Yang et al., 2014, 2016), and that action-based benefits during encoding can be displayed in children with autism spectrum disorder (Wojcik et al., 2011) and older adults with Alzheimer’s disease (Charlesworth et al., 2014). In line with arguments concerning similar manipulations in the episodic long-term memory literature (e.g., Cohen, 1981), the resulting benefits may be relatively automatic and non-strategic in nature. The possible automaticity and independence from effortful control processes of such benefits, along with potential increases in task engagement that such manipulations may encourage and explain why performance is improved in children with ADHD despite their wider profile of cognitive impairments.

The ADHD children were a convenience sample, which does introduce certain limitations. First, the majority of the ADHD sample were on medication during testing. However, it should be noted that there were no differences in attention between those who were medicated and those who were not. To exclude the influence of medication, future work should replicate and extend this study with unmedicated samples or prohibit the ADHD sample from taking medication with sufficient time prior to testing for medication effects to wash out. A second limitation is that the ADHD group consisted of children with both combined and inattentive subtypes. Although there were no differences in performance between the inattentive and combined subtypes, the sample sizes were relatively small. The conclusions drawn here are therefore preliminary and necessitate further exploration. Finally, a third limitation is that we did not exclude children with comorbid disorders, such as dyslexia and language impairment. Language problems are often comorbid with ADHD (Willcutt and Pennington, 2000; Germano et al., 2010; Hawkins et al., 2016), with comorbidity rates of between 18 and 45% for dyslexia (Germano et al., 2010), and 40–45% for language impairment (Tirosh and Cohen, 1998; Helland et al., 2016). Children with comorbid language problems have difficulties in encoding, retaining, and retrieval verbal information (Archibald and Gathercole, 2006) and so may be more impaired in primarily verbal conditions (e.g., spoken presentation or verbal recall conditions). This should be an interesting area for future investigation and has implications for comorbid language disorders when instructions are to be presented or recalled verbally.

This study has important practical implications concerning the ability to follow instructions in different developmental groups. The classroom is a predominantly verbal environment, which places heavy demands on children to retain spoken or written instructions either overtly or sub-vocally. The current data suggest that teacher demonstration may improve the retention of important information both for children with and without developmental difficulties. The benefits observed in both groups when they knew they had to act out the instruction sequences suggests that telling children to plan for action could also enhance the encoding, retention and successful implementation of classroom instructions. To our knowledge, this is the first study to directly assess FI skills in children with ADHD and show they have substantial impairments. This provides a possible target for training and intervention. Future work should continue to explore how to optimize the demonstration methodology in practical contexts.

## Ethics Statement

Experimental procedures were approved by the Institutional Review Board of the Institute of Psychology, Chinese Academy of Sciences and the Peking University Sixth Hospital. The experiment was carried out in accordance with the approved guidelines. Informed consent was obtained from parents/carers at the start of the testing. During the stage of data analyses and data archive, demographic and clinical information was kept confidential.

## Author Contributions

TY generated the idea, designed the study, analyzed the data, and wrote the paper. RA interpreted the data and wrote the paper. JH contributed significantly to the interpretation of the findings and writing of the paper. RC contributed to the design of the study and interpretation of the findings. All authors read and commented the final version of the paper.

## Conflict of Interest Statement

The authors declare that the research was conducted in the absence of any commercial or financial relationships that could be construed as a potential conflict of interest.

The reviewer KH and the handling Editor declared their shared affiliation, and the handling Editor states that the process nevertheless met the standards of a fair and objective review.
