# Supplementary material for: Impaired Memory for Instructions in Children with Attention-Deficit Hyperactivity Disorder Is Improved by Action at Presentation and Recall
Source: Front Psychol. 2017 Jan 24;8:39. doi: 10.3389/fpsyg.2017.00039 (PMC5258743; doi:10.3389/fpsyg.2017.00039)
Supplement: Supplementary file 1 [file Data_Sheet_1.pdf]

Supplementary Material

Descriptive statistics and group comparisons of following instruction performances between the two ADHD subtypes and between the medicated and unmediated group are shown in Table S1. Only two children in the ADHD group belong to the hyperactive/impulsive subtype, therefore their performances were not compared with other subtypes. There were no significant differences in performance across all conditions of the FI span task between the inattentive and combined subtype of ADHD (*ps* > *.05*), or between the medicated and unmedicated ADHD groups (*ps* > *.05*).

Table S1

Performances of following instructions in different subtypes and medication groups of ADHD

	Inattentive (N = 10)		Combined (N = 12)		Group Comparison		
	Means	*SD*	*Means*	*SD*	*t*	*p*	*Cohen's d*
Spoken-Verbal recall	31.20	13.98	27.42	9.98	0.74	0.468	0.33
Spoken-Enacted recall	42.00	8.76	39.67	9.13	0.61	0.550	0.27
Demo-Verbal recall	37.50	19.28	30.00	9.02	1.13	0.280	0.54
Demo-Enacted recall	37.00	8.63	38.67	18.00	-0.27	0.780	0.12
	Unmedicated (N = 6)		Medicated (N = 18)		*t*	*p*	*Cohen's d*
Spoken-Verbal recall	26.00	9.86	30.50	12.40	-0.80	0.430	0.40
Spoken-Enacted recall	38.00	10.81	41.50	8.79	-0.80	0.432	0.39
Demo-Verbal recall	32.17	6.97	34.17	15.91	-0.30	0.771	0.15
Demo-Enacted recall	36.83	7.81	39.33	15.50	-0.38	0.711	0.18
